# Photocatalytic Degradation of Antibiotics via Exploitation of a Magnetic Nanocomposite: A Green Nanotechnology Approach toward Drug-Contaminated Wastewater Reclamation

**DOI:** 10.1021/acsomega.3c08116

**Published:** 2024-02-06

**Authors:** Noor Zulfiqar, Raziya Nadeem, Othman AI Musaimi

**Affiliations:** † Department of Chemistry, Faculty of Science, University of Agriculture, Faisalabad 38000, Pakistan; ‡ Department of Chemistry, Faculty of Science, University of Agriculture, Faisalabad 38000, Pakistan; § School of Pharmacy, Faculty of Medical Sciences, Newcastle upon Tyne NE1 7RU, U.K.; ∥ Department of Chemical Engineering, Imperial College London, London SW7 2AZ, U.K.

## Abstract

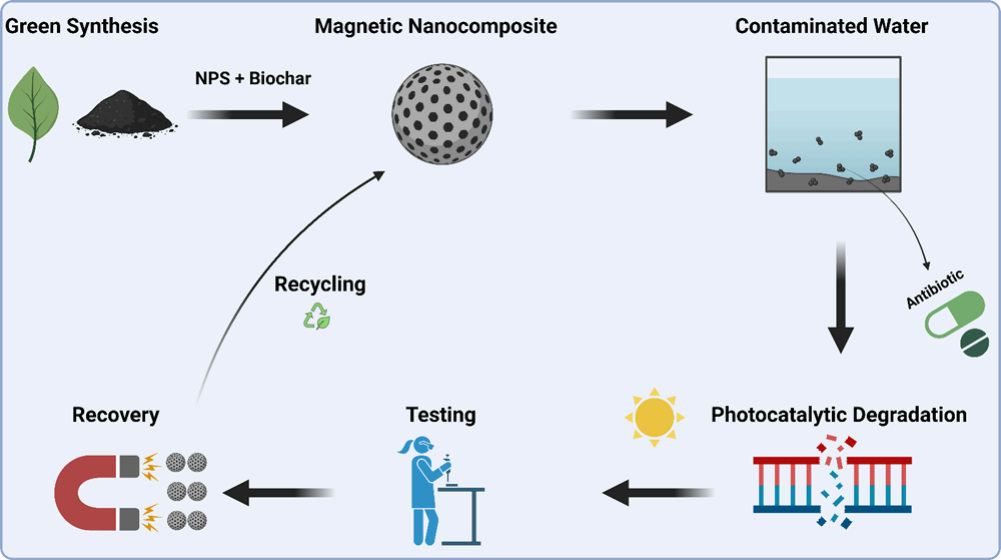

In the quest for eco-conscious innovations, this research was designed for the sustainable synthesis of magnetite (Fe_3_O_4_) nanoparticles, using ferric chloride hexahydrate salt as a precursor and extract of *Eucalyptus globulus* leaves as both a reducing and capping agent, which are innovatively applied as a photocatalyst for the photocatalytic degradation of antibiotics “ciprofloxacin and amoxicillin”. Sugar cane bagasse biomass, sugar cane bagasse pyrolyzed biochar, and magnetite/sugar cane bagasse biochar nanocomposite were also synthesized via environmentally friendly organized approaches. The optimum conditions for the degradation of ciprofloxacin and amoxicillin were found to be pH 6 for ciprofloxacin and 5 for amoxicillin, dosage of the photocatalyst (0.12 g), concentration (100 mg/L), and irradiation time (240 min). The maximum efficiencies of percentage degradation for ciprofloxacin and amoxicillin were found to be (73.51%) > (63.73%) > (54.57%) and (74.07%) > (61.55%) > (50.66%) for magnetic nanocomposites, biochar, and magnetic nanoparticles, respectively. All catalysts demonstrated favorable performance; however, the “magnetite/SCB biochar” nanocomposite exhibited the most promising results among the various catalysts employed in the photocatalytic degradation of antibiotics. Kinetic studies for the degradation of antibiotics were also performed, and notably, the pseudo-first-order chemical reaction showed the best results for the degradation of antibiotics. Through a comprehensive and comparative analysis of three unique photocatalysts, this research identified optimal conditions for efficient treatment of drug-contaminated wastewater, thus amplifying the practical significance of the findings. The recycling of magnetic nanoparticles through magnetic separation, coupled with their functional modification for integration into composite materials, holds significant application potential in the degradation of antibiotics.

## 1 Introduction

Water is the main element that supports life on earth. It should be pollutant-free for drinking as well as for residential and industrial uses. In developing countries around the world, treatment of water pollution and the protection of the environment are taken as special concerns. The contaminants that are present particularly in water mainly consist of pharmaceutical drugs, heavy metals, organic pollutants like detergents, biomaterials, and inorganic pollutants.^1,2^ When these pollutants are left untreated, they may become a threat to the environment and living beings, which makes it necessary to treat the unhygienic water.^3^

Pharmaceutical drugs (toxic antibiotics) are the dominating contributors to water contamination, released from various sources such as discharge of expired medication in the environment and pharmaceutical projects.^4^ Emergency clinics are a great source to discharge pharmaceutical drugs in the environment from different assets.^5^ Fluoroquinolone (FQ) antibiotics, e.g., ciprofloxacin (CIP), cannot be entirely metabolized in humans and animals, as reported in the literature that 60–90% of these antibiotics are metabolized,^6^ discharged into the environment through feces,^6−8^ and also, these antibiotics cannot be efficiently confiscated using existing water treatment methodologies. Consequently, FQs are released into the ecosystem and become developing environmental pollutants.^8^ These medications may cause a threat to human health and the ecosystem.^9^ In contrast to FQ antibiotics, β-lactams, e.g., amoxicillin (AMX), belong to a group of antibiotics known as narrow-spectrum antibiotics. These antibiotics function as bacteriostatic agents by inhibiting the production of the peptidoglycan cell wall in bacteria. They exhibit particularly strong effectiveness against Gram-positive bacterial genera such as Streptococcus, Gonococcus, and Staphylococcus. However, it is important to note that the complete metabolism of β-lactam antibiotics, such as AMX, is challenging for both humans and animals. Consequently, these compounds are often excreted into aquatic ecosystems, posing potential ecological concerns, for example, municipal wastewater, rivers, streams, lakes, and seawater.^10^

Application of green nanotechnology could likewise help to meet the requirement for pure natural drinking water through the quick treatment of pollutants in water. Green chemistry deals with less hazardous, nontoxic, and environmentally friendly chemical substances.^11,12^ In green materials, different compounds exist that act as reducing agents and also capping or stabilizing agents for NPs.^6^^,^^10^ The green synthesis of nanoparticles (NPs) is combined and reestablished using a few methodical techniques. These days, researchers have used plant biowastes for the sustainable fabrication of NPs because the extrication of plant materials is harmless and viable.^12^

In the context of water treatment, traditional methods often involve approaches such as chemical coagulation, sedimentation, and advanced oxidation processes for the removal of contaminants. For example, chemical coagulation relies on the addition of chemicals to form precipitates that can be removed through sedimentation.^13^ While effective, these traditional methods may have drawbacks such as the generation of chemical sludge,^14^ slower process, and nonecofriendly and high-energy consumption, contributing to higher operational costs.^15^ In comparison, our research, employing green-synthesized magnetic nanoparticles to engineer an innovative magnetic nanocomposite for photocatalytic antibiotic degradation not only showcases improved efficiency but also offers potential cost benefits by utilizing ecofriendly materials and promoting regeneration for reuse. The contrast highlights the potential of our approach to address both efficiency and cost-effectiveness in water treatment compared to certain traditional methods.^16^

In the following research, *Eucalyptus globulus* leaf extract is used as a capping agent or reducing agent for the synthesis of magnetic NPs. Magnetite NPs are colloidal iron oxide (Fe_3_O_4_) materials that display superparamagnetic characteristics at room temperature. Their size, nontoxic nature, and superparamagnetic characteristics make them fascinating for applications in numerous fields, e.g., biosensors, catalysis, magnetic separations, ferro fluids, and magnetic resonance imaging.^17^ Studies on magnetite NPs have revealed that CIP, which belongs to the fluoroquinolone class of drugs, has excellent interaction with the metal oxide NPs.^18,19^ Similarly, degradation of AMX using green-synthesized iron oxide NPs was also reported.^20^

Biochar is a material that is highly rich in the carbon content and created during thermal decomposition of wasted organic materials by a controlled supply of oxygen (also called pyrolysis) at generally low temperatures (<700 °C).^21,22^ In this exploration work, we have applied the biochar synthesized using sugar cane bagasse biomass, which was air-dried and warmed in a clump-type pyrolysis heater at 600 °C for 2 h.^22^ The composite is a mixture of two constituents with distinctive properties to give a material of characteristics better than both constituents taken separately. Components of the nanocomposite have a high surface-to-volume ratio due to their small size and surface activity.^23^ A magnetic nanocomposite typically comprises a combination of magnetic NPs, embedded within or dispersed throughout a matrix material (biochar). The magnetic nanocomposite generally retains high adaptability, for example, they show unobtrusive development, small size of particles, large surface area-to-volume ratio, bioadsorption, and photocatalytic advantages.^23,24^ The magnetic nanocomposite exhibits a high degree of adaptability by easily adjusting its properties to suit diverse wastewater compositions. The magnetic component facilitates the efficient recovery of the nanocomposites after treatment, enhancing their reusability. Additionally, their bioadsorption capacity enables effective removal of microcontaminants of various ranges, such as heavy metals and pharmaceutical pollutants, through surface interactions with biological entities integrated into the composite structure.^25^ Furthermore, the incorporation of photocatalytic materials enhances their ability to degrade persistent pollutants under light irradiation, contributing to a comprehensive and sustainable wastewater treatment solution.^26^ This multifaceted approach underscores the potential of magnetic nanocomposites as advanced, viable, and versatile tools in addressing the complex challenges associated with wastewater remediation.^14,26,27^

**Figure 1:**
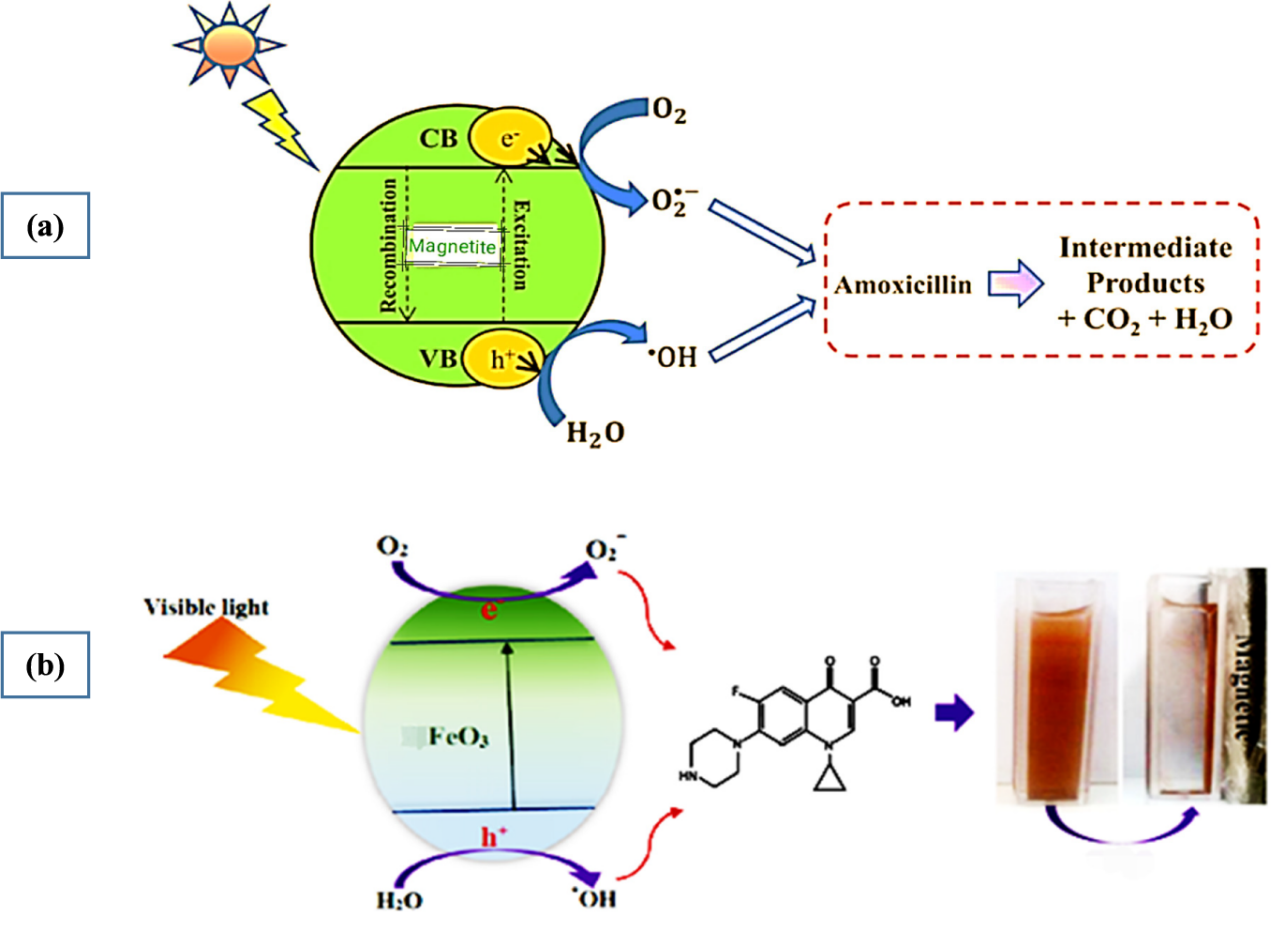
Mechanism of photocatalytic degradation for antibiotics (a) amoxicillin and (b) ciprofloxacin. Reprinted with permission from refs (34) and (36). Copyright 2020 Global Journal of Environmental Science and Management and 2019 Scientific Reports.

Advanced oxidation techniques, for example, the Fenton oxidation process and photocatalytic degradation, are frequently used for degradation of antibiotics, but developing huge volumes of iron slurry is a significant issue of the Fenton oxidation process, so photocatalytic degradation is the preferred method for the degradation of antibiotics.^28−31^ In the photocatalytic degradation of antibiotics by magnetic nanocomposites, the incident light triggers the excitation of electrons from the valence band to the conduction band within the catalyst, generating electron–hole pairs (e^–^/h^+^). These electron–hole pairs then act as initiators in an oxidative degradation process. Concurrently, interactions with water molecules present in the reaction medium lead to the formation of highly reactive hydroxyl radicals. These radicals effectively oxidize and degrade the antibiotic molecules adsorbed onto the surface of the nanocomposite through oxidative and radical-driven reactions, ultimately leading to the transformation of the antibiotics into less harmful byproducts. The magnetic component enables easy separation and recovery of the nanocomposite catalyst from the treated solution using an external magnetic field, enhancing the reusability and practical applicability of the photocatalytic system.^29−35^ The mechanism of photocatalytic degradation for both antibiotics CIP and AMX is shown in Figure 1. Fe_3_O_4_ creates a reactive species in the presence of UV light, for example, hydroxyl radicals (HO·) as indicated by the accompanying reactions:
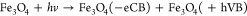
1
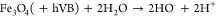
2
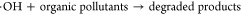
3

In comparison to similar works reported in the literature,^37^ this study stands out in several key aspects. First, the use of *E. globulus* leaf extract as an iron precursor for the green synthesis of magnetic nanoparticles introduces a novel and sustainable approach that sets it apart from traditional and conventional methods, e.g., precipitation, adsorption, and membrane.^38^ This ecofriendly synthesis method aligns with the principles of green chemistry,^39^ contributing to the development of environmentally friendly water treatment technologies. Moreover, loading of Fe_3_O_4_ NPs on to a matrix (biochar) with subsequent incorporation of biochar derived from sugar cane bagasse biomass as a matrix material in the magnetic nanocomposite distinguishes this work.^40^ Biochar, known for its high carbon content and created through controlled pyrolysis, introduces unique properties to the composite, potentially enhancing its adsorption capabilities.^2^

Additionally, the application of this magnetic nanocomposite for the photocatalytic degradation of antibiotics, specifically targeting pharmaceutical drugs like ciprofloxacin and amoxicillin, showcases a focused, innovative, and relevant approach to water treatment. The study’s exploration of various parameters, including irradiation time, pH levels, and initial concentration, adds a comprehensive dimension, providing a deeper understanding of the factors influencing the effectiveness of the treatment process. Finally, the use of three distinct kinetic models for analysis of pseudo-first-order, pseudo-second-order, and the Behnajady–Modirashahla–Ghanbery (BMG) models demonstrates a rigorous and systematic investigation, contributing to the reliability and applicability of the findings.^41^ In summary, the combination of green synthesis, unique composite materials, targeted pollutant degradation, and a comprehensive analytical approach distinguishes this work from similar studies in the literature.^19,28,42,43^

## 2 Materials and Methods

### 2.1 Materials and Chemicals

Leaves of **E. globulus** were collected from precincts of the University of Agriculture, Faisalabad for the synthesis of extract. Sugar cane bagasse was collected from a local marketplace of Faisalabad for the preparation of biomass and biochar. In this research, we employed specific chemicals, namely, ferric chloride hexahydrate (FeCl_3_·6H_2_O), ferrous chloride tetrahydrate (FeCl_2_·4H_2_O), and sodium hydroxide (NaOH), which were procured from Aldrich Chemicals. To ensure the purity of our reagents, we used sterile distilled water sourced from the biochemistry laboratory, with a conductivity measurement of 1 μS/cm. All the chemicals used in this study met analytical grade standards and did not require further purification. We meticulously prepared our solutions using deionized water, and all dilutions were carried out using double-distilled water.

### 2.2 Synthesis of the Magnetic Photocatalyst

The synthesis of **E. globulus** leaf extract involved collecting 20–40 g of leaves, washing them with deionized water, drying at room temperature, soaking in distilled water, and then shaking them in an orbital shaker. The homogenized extract was filtered and stored at low temperature for further use in the phytoassisted synthesis of FeO NPs.^44^ The green methodology employed in this study involved the application of **E. globulus** leaf extract as a multifunctional agent. This extract served as a stabilizing, reducing, and capping agent during the preparation of magnetite NPs. Notably, its presence played a pivotal role in preventing the agglomeration of magnetic NPs throughout the synthesis process. FeCl_3_·6H_2_O was used as the iron precursor. The process involved adding 13.9 g of the reducing agent and 6.75 g of FeCl_3_·6H_2_O and stirring for 4 h at 80 °C. Sodium hydroxide was added, and the black powdered magnetite NPs were dried in a drying oven for 2–3 h.^45,46^

**Figure 2:**
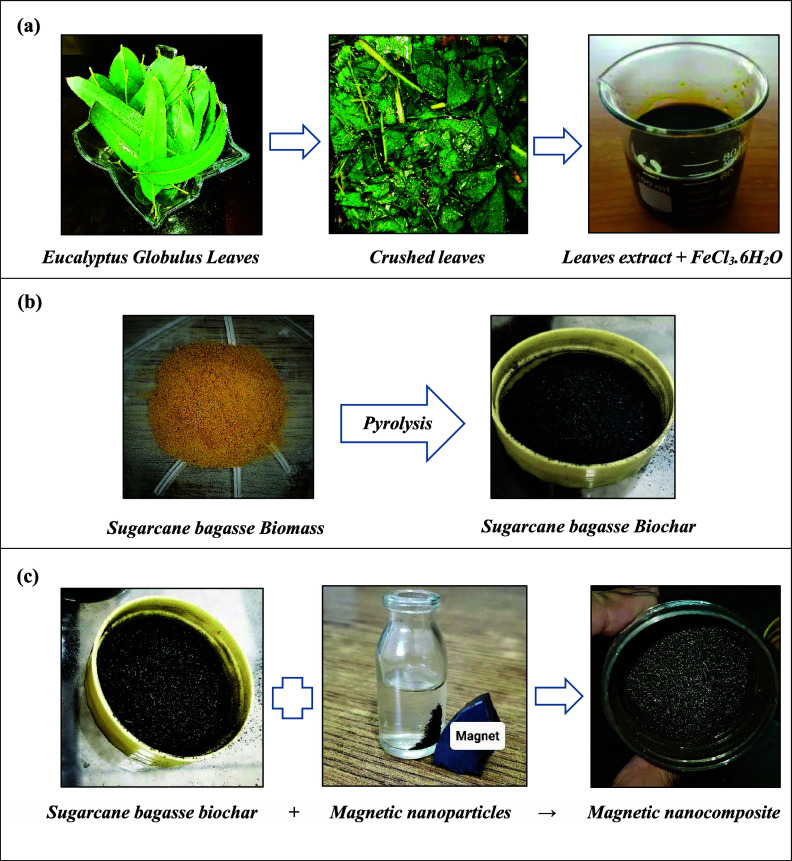
(a) Synthesis of magnetite NPs from **E. globulus** leaf extract, (b) syntheses of sugar cane bagasse biomass and sugar cane bagasse biochar, and (c) synthesis of the magnetic nanocomposite.

Sugar cane bagasse was collected from a local market and household waste and washed, cleaned, dried, crushed, and converted into biomass, and then, the biomass was pyrolyzed at 530 °C for 2 h to synthesize biochar. The synthesized biochar was stored for the synthesis of the nanocomposite.^2^ For preparation of magnetic nanocomposites, 1 g of biochar of sugar cane bagasse and 0.2 g of magnetic NPs were dispersed in 100 mL of distilled water by sonication and then stirred constantly at a rate of 200 rpm for 4 h. After this, the magnetic nanocomposite was separated by external magnetic force and ultimately dried out completely in an oven at about 80 °C.^21,47^ The synthesis of magnetite NPs from **E. globulus** leaf extract, the syntheses of sugar cane bagasse biomass and sugar cane bagasse biochar, and the synthesis of the magnetic nanocomposite are shown in Figure 2.

### 2.3 Characterization of the Photocatalyst

The prepared photocatalyst was characterized using various techniques, including UV-visible spectrophotometry for verifying the presence of magnetic NPs, Fourier transform infrared (FT-IR) for identifying functional groups, and scanning electron microscopy (SEM) for studying surface morphology before and after the photocatalytic experiment. The photocatalyst was prepared by dispersing magnetic NPs in biochar through ultrasonication for 3 h and subsequent drying followed by a 2 min microwave irradiation to improve exfoliation. This comprehensive characterization provided valuable insights into the physicochemical properties and structural features of the photocatalyst, which are essential for its potential application in environmental remediation and wastewater treatment.

### 2.4 Preparation of Solution for Photocatalytic Degradation

In the context of our research investigation, we meticulously formulated a high-concentration solution comprising 1000 mg/L AMX and CIP, accurately dissolving them in distilled water. Employing a precisely engineered SCB/Fe_3_O_4_ nanocomposite, we harnessed its remarkable capabilities to effectuate the degradation of these pharmaceutical compounds from an aqueous milieu. Subsequently, we conducted a rigorous quantitative assessment, ascertaining the concentration of these drugs both prior to and subsequent to their exposure to this advanced nanomaterial-based degradation process and employing the precision of a UV–vis spectrophotometer to gauge the extent of their transformation. Moreover, solutions were prepared using standard solution and by applying the formulas of dilution.^48^

### 2.5 Optimization of Process Parameter Methodology

#### 2.5.1 Impact of the pH Value

A photocatalytic experiment was conducted in test tubes containing 100 mg/L solution of AMX. Initially, the pH of the prepared solutions was maintained in the range of 2–11 using 0.1 M HCl and 0.1 M NaOH solutions. The same process was used for another antibiotic CIP.^31^

#### 2.5.2 Concentration of Antibiotics

The effect of the initial concentration of antibiotics was studied. The catalytic degradation experiment was conducted using stock solutions of different initial concentrations of pharmaceutical drugs of 10, 20, 40, 60, 80, and 100 mg/L.^34^

#### 2.5.3 Quantity of the Photocatalyst

Degradation of antibiotics was investigated using different photocatalyst dosages of 0.05, 0.07, 0.09, and 0.1 g in 100 mg/L solution of each drug.^49^

#### 2.5.4 Effect of Experimental Duration

The photocatalytic experiment was carried out at different time intervals (30, 60, 120, 180, and 240 min) using the same pH, catalyst dosage, and initial concentration of solution.^50^

#### 2.5.5 Degradation Efficiency or % Removal

The % degradation of drugs was given as^51^
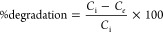
4where *C*_i_ is the initial concentration of the drug in solution (mg/L) and *C*_e_ is the final concentration of drugs in solution (mg/L).

(52)

#### 2.5.6 Kinetic Models for Degradation of Drugs

Kinetic modeling stands as a key determinant in ascertaining the efficacy of drug degradation processes. Kinetic models for drug degradation are the first-order, second-order, and BMG models. These models serve as valuable tools in validating and rationalizing the experimental data obtained during drug degradation investigations. Different mathematical models were derived to utilize the results in real applications and also to stimulate the reaction kinetics.^53^ These are given as

##### First-Order Kinetics


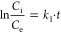
5

##### Second-Order Kinetics


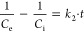
6

##### Behnajady–Modirashahla–Ghanbery


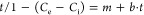
7where *C*_e_ is the drug concentration at time “*t*”, *C*_i_ is the drug concentration at time “0”, *k*_1_ is the rate constant for the first-order reaction, *k*_2_ is the rate constant for the second-order reaction, *t* is time (s^–1^), and *m* and *b* are constants of the BMG model relating to oxidation capacities and reaction.^54^

#### 2.5.7 Statistical Investigation

All of the results were statistically analyzed using simple linear regression.^55^

## 3 Results and Discussion

The study’s discussion emphasizes the potential of a green nanotechnology approach for effective wastewater reclamation, which can help in addressing the escalating concern of antibiotic pollution in water bodies. The use of a magnetic nanocomposite as a photocatalyst offers several advantages, such as easy separation from the treated water due to its magnetic properties, reusability, and minimal generation of harmful byproducts. The results demonstrated that the magnetic nanocomposite exhibited a high efficiency in degrading a wide range of antibiotics commonly found in drug-contaminated wastewater. Through a series of photocatalytic experiments, the researchers observed significant degradation rates of antibiotics, leading to the breakdown of their molecular structures.^30,34,56^

### 3.1 Characterization of the Photocatalyst

#### 3.1.1 Characterization of Fe_3_O_4_ NPs

**Figure 3:**
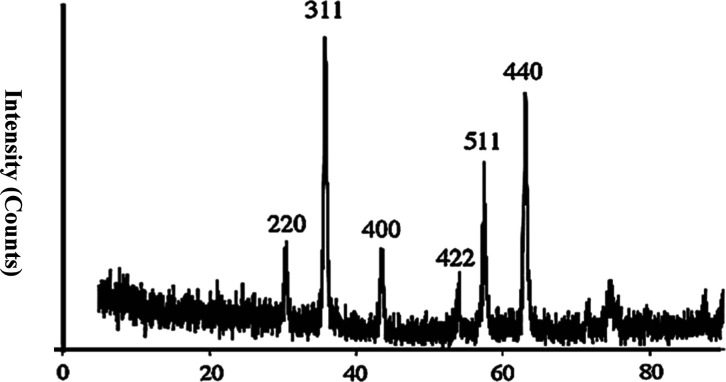
Confirmation of the X-ray diffraction pattern for Fe_3_O_4_ NPs by analysis of the corresponding *hkl* indices.

The assessment of the purity of the synthesized Fe_3_O_4_ NPs was conducted through an X-ray diffraction (XRD) analysis. Figure 3 presents the XRD pattern acquired for the resultant powders. The observed XRD spectrum exhibited discernible peaks corresponding to the crystallographic indices (220), (311), (400), (422), (511), and (440), which are indicative of the presence of nanoscale particles of Fe_3_O_4_. The highest intensity peak is at 38.22°. These findings are in excellent agreement with previously reported results pertaining to Fe_3_O_4_ NPs, affirming the integrity and consistency of our synthesized material.^57^ The mean crystallite size (denoted as *D*) was determined through the application of the Debye–Sherrer formula, expressed as *D* = *K*λ/β cos θ. In this equation, *K* represents the Sherrer constant, λ signifies the X-ray wavelength, β denotes the width of the peak at half-maximum, and θ corresponds to the Bragg diffraction angle. This formula was employed to ascertain the average crystallite size, which is a pivotal parameter in our analytical investigation. As a result, it was discovered that the average size of the crystallite for the powdered sample was approximately 24 nm.

**Figure 4:**
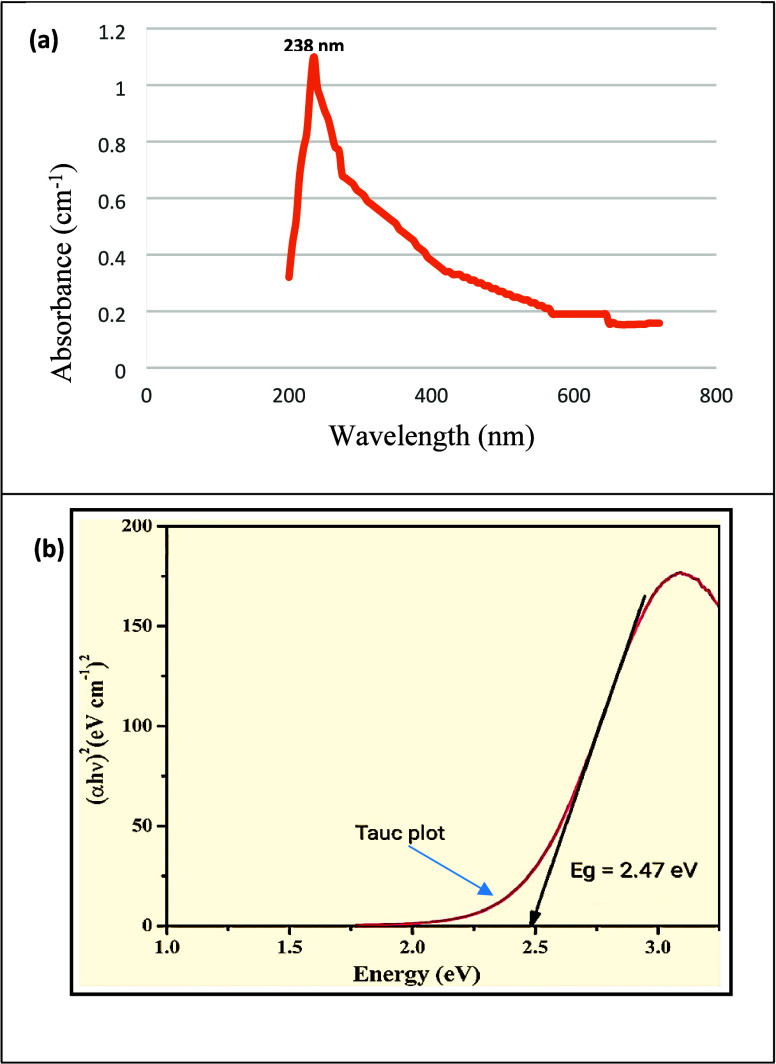
(a) UV spectra and (b) Tauc plot of Fe_3_O_4_ NPs.

For performing UV analysis, Fe_3_O_4_ NPs were subjected to 30 min of sonication in deionized water, resulting in a clear colloid solution. Deionized water served as the reference to maintain spectral purity, minimizing interference from ions and impurities as well as enhancing the reliability of the UV absorption spectroscopy results. The absorbance spectrum displayed an absorption peak at 238 nm within the UV wavelength range (Figure 4a) displaying the UV spectral graph of magnetic nanoparticles with absorbance plotted against the wavelength of incident light. This plot is crucial in UV analysis as it reveals the material’s absorption characteristics. The distinctive peaks and absorption edge observed at 238 nm in the spectrum provide valuable insights into the nanoparticles’ electronic transitions and, when analyzed using methods like the Tauc plot,^58^ facilitate the determination of the direct band gap energy, contributing to a comprehensive understanding of their optical properties (Figure 4b). Remarkably, black-colored Fe_3_O_4_ NPs demonstrated broad absorption in this specific region.^59,60^

In this study, the band energy of magnetic nanoparticles was investigated by using UV absorption spectroscopy, revealing a distinct absorption edge at 238 nm. The absorption edge marks the initiation of electronic transitions within the material. Employing the Tauc plot method, the square of the absorption coefficient (α*h*ν)^2^ is plotted against the energy of incident photons (*h*ν). In constructing the Tauc plot, linear regression software was employed to determine the straight-line fit for the relationship between (α*h*ν)^1/*n*^ and *h*ν, and in the Tauc plot, different values of *n* where *n* = 1/2, 3/2, and 2 were considered, and the optimal fit value was investigated specifically for the case where 1/*n* equals 2. This value was assigned to represent a direct allowed transition, and the resulting Tauc plot exhibited a linear region in the high-energy range.^61^ This behavior suggests that the magnetic nanoparticles exhibit a direct band gap semiconductor nature. By extending the linear part of the plot to the *x* axis, we found that the experimentally measured band gap energy was around 2.47 eV. This value is in line with a literature value of 2.52 eV, confirming the accuracy of our experimental findings.^62^ The position of the valence band and conduction band was determined quantitatively as follows:

*X* is the 1.6 eV electron affinity for magnetic nanoparticles, *E*_e_ is the 0.1 eV energy level of the free electron for magnetic nanoparticles, and *E*_g_ is the 2.47 eV energy gap for magnetic nanoparticles.

For the conduction band (*E*_CB_):
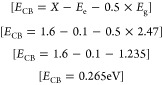
8

For the valence band (*E*_VB_):
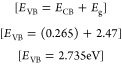
9

Simultaneously, the positions of the valence band (VB) and conduction band (CB) were identified. The VB was situated at an energy level of approximately 2.735 eV, while the CB was positioned at approximately 0.265 eV. Figure 4 represents the UV spectra and Tauc plot of Fe_3_O_4_ NPs.

The study assumes a linear relationship between the square of the absorption coefficient and photon energy in the Tauc plot method, indicating direct band gap semiconductor properties in magnetic nanoparticles. The extension of the linear region to the *x* axis for band gap determination assumes homoscedasticity.

#### 3.1.2 Scanning Electron Microscopy (SEM), Transmission Electron Microscopy (TEM), and Energy-Dispersive X-ray (EDX) Analyses

**Figure 5:**
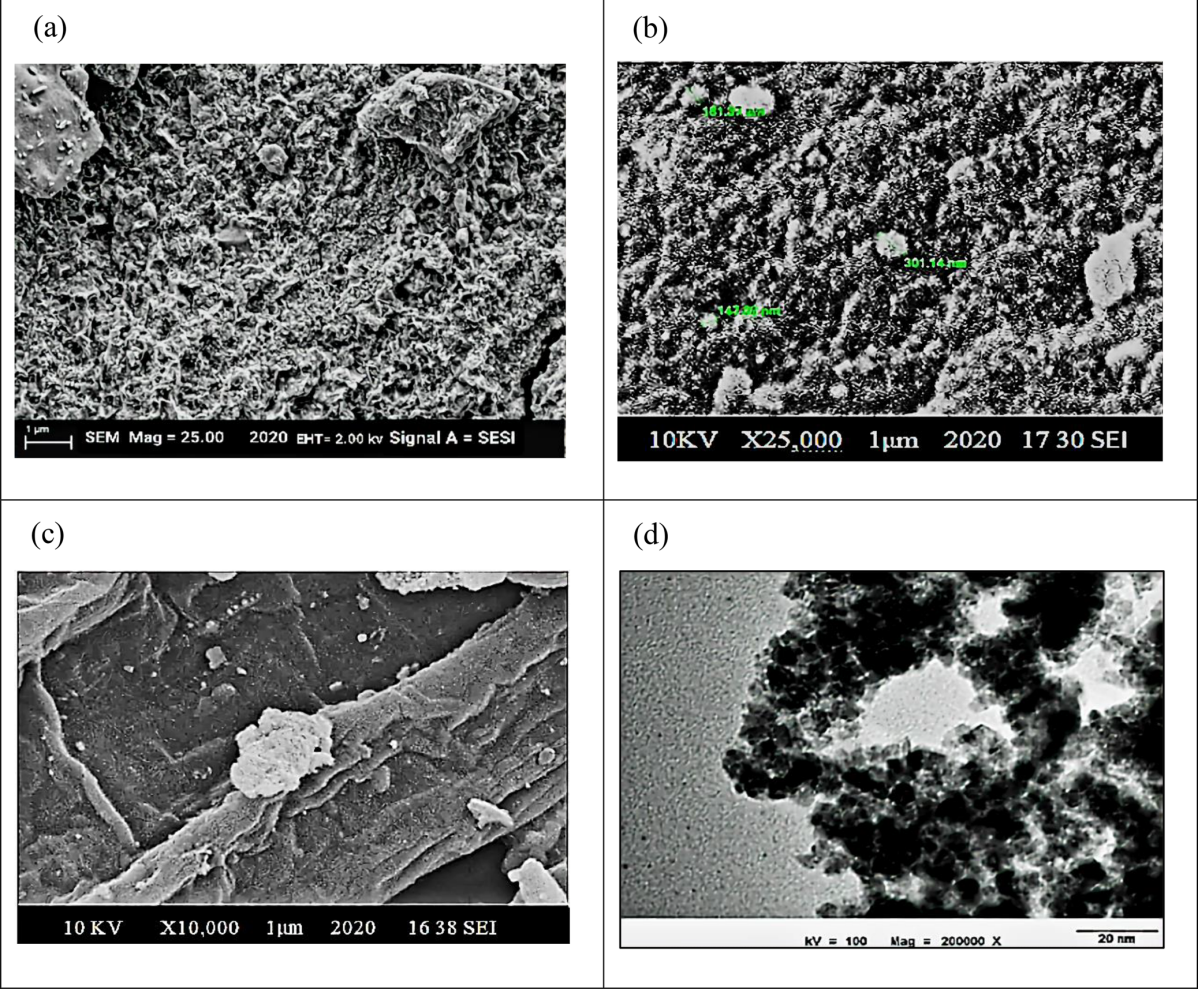
Scanning electron microscopy (SEM) images of Fe_3_O_4_ NPs and magnetite/SCB biochar (a–c) and (d) size and dispersion of Fe_3_O_4_ NPs in ethanol solution as seen through transmission electron microscopy.

The application of plant-derived compounds as capping agents has resulted in the synthesis of remarkably stable NPs characterized by their exceptional refinement. These magnetite NPs, skillfully crafted through this method, exhibited semi-spherical morphologies and demonstrated a remarkably narrow and uniform size distribution in the range of 100–200 nm; the semi-spherical shape optimizes light absorption, enhancing the generation of electron–hole pairs upon exposure to UV or visible light. The nanocomposite’s uniform size distribution ensures consistent charge carrier separation, minimizing recombination. The SEM image of the magnetite/SCB biochar nanocomposite is shown in Figure 5. White brightened semi-spherical magnetite NPs are also clearly observed on the pyrolyzed sugar cane bagasse surface.

The outcomes derived from the SEM analysis underscore the significant influence of the iron salt-to-leaf extract ratio on the grain sizes of the NPs, even when identical reaction conditions were maintained. The SEM images vividly illustrate a rich tapestry of microstructural attributes, alongside a spectrum of diverse morphologies exhibited by the synthesized Fe_3_O_4_ nanopowder. To comprehensively characterize the synthesized Fe_3_O_4_ NPs, TEM emerged as the preferred analytical tool, as depicted in Figure 5. The images revealed an ensemble of exceedingly diminutive NPs with an average diameter of 5.49 ± 1.35 nm. The distinctive cluster-like configuration observed may be attributed to the interaction of environmental molecules adhering to nanoparticle surfaces, seeking to establish an equilibrium of intermolecular forces. The TEM-observed cluster-like morphology in the magnetic nanocomposite enhances light absorption, optimizing charge carrier dynamics and fostering synergistic interactions. This configuration improves the efficiency of the photocatalytic degradation mechanism, providing additional active sites for enhanced adsorption and pollutant removal in drug-infested wastewater.

**Figure 6:**
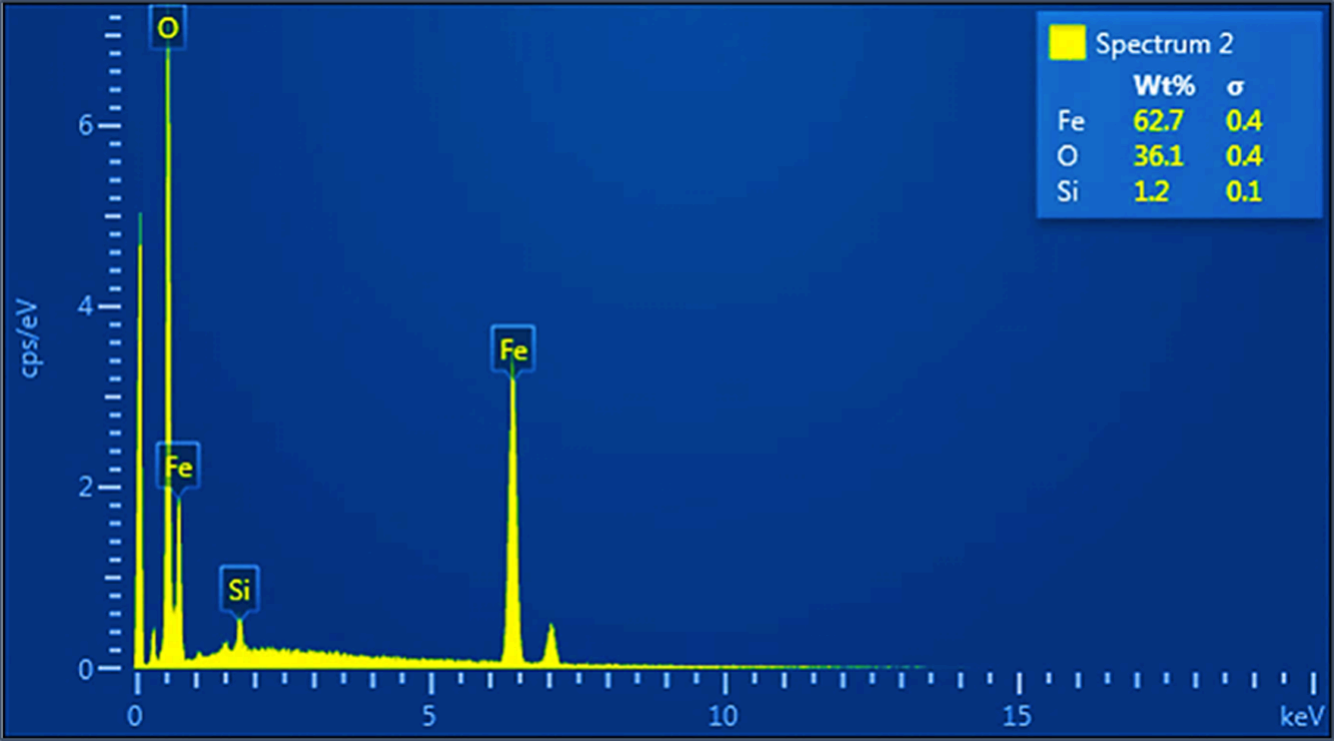
EDX analysis image of Fe_3_O_4_ NPs.

EDX analysis is vital for quality control as it precisely identifies and quantifies impurities in materials, ensuring their purity. The technique, providing detailed elemental composition information, aids in optimizing synthesis processes and enhancing the overall performance and reliability of the final product.^63^ The composition of the as-synthesized NPs with magnetic properties is shown by the EDX spectrum analysis displayed in Figure 6. The EDX spectra revealed significant Fe and O peaks. According to the study, the compositions are 62.7% for Fe, 36.1% for O, and 1.2% for Si. The EDX analysis of all the Fe_3_O_4_ NPs is shown in Figure 6. The analysis of the EDX spectrum revealed the presence of both iron (Fe) and oxygen (O) within their respective spectra. Intriguingly, an additional observation pertains to the detection of foreign substances within the spectrum. This phenomenon may be accredited to the potential impurity within the sample under investigation.

#### 3.1.3 Magnetic Properties (VSM)

Vibrating sample magnetometry (VSM) and superconducting quantum interference device (SQUID) measurements were employed to analyze the magnetic properties of nanoparticles, such as saturation magnetization, coercivity, and remanence.^64^ Analysis of the magnetic nanoparticles through vibrating sample magnetometry (VSM) measurements yielded important findings regarding their magnetic characteristics. The hysteresis loop, derived from the VSM analysis, showed a saturation magnetization (Ms) of 87.95 emu/g at 15 kOe and a remnant magnetization (Mr) of 12.49 emu/g. This confirmed the vigorous magnetic behavior of the nanoparticles. The presence of hysteresis was found by Prijic et al.^65^ A coercivity (Hc) value of 123.94 Oe indicated their sensitivity to external magnetic fields, with the observation of reversible magnetization transitions.

**Figure 7:**
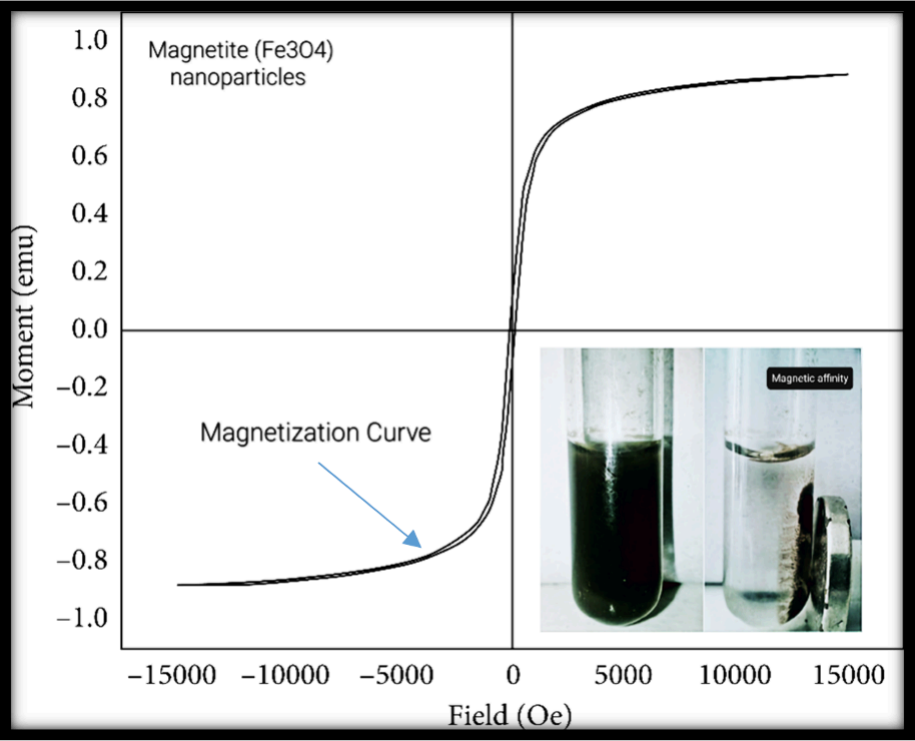
Vibrating sample magnetometry (VSM) of magnetic nanoparticles.

The VSM data supported the superparamagnetic nature of nanoparticles. These results aligned with the nanoparticles’ nanoscale dimensions, consistent with the expected enhanced magnetic behavior at reduced sizes. The outcomes of the VSM analysis offer crucial insights for understanding the response of magnetic nanoparticles to external magnetic fields. This understanding is pivotal for their potential applications in diverse areas, such as biomedicine and data storage. Figure 7 depicts the vibrating sample magnetometry (VSM) of magnetic nanoparticles.

#### 3.1.4 FT-IR Spectroscopy

**Figure 8:**
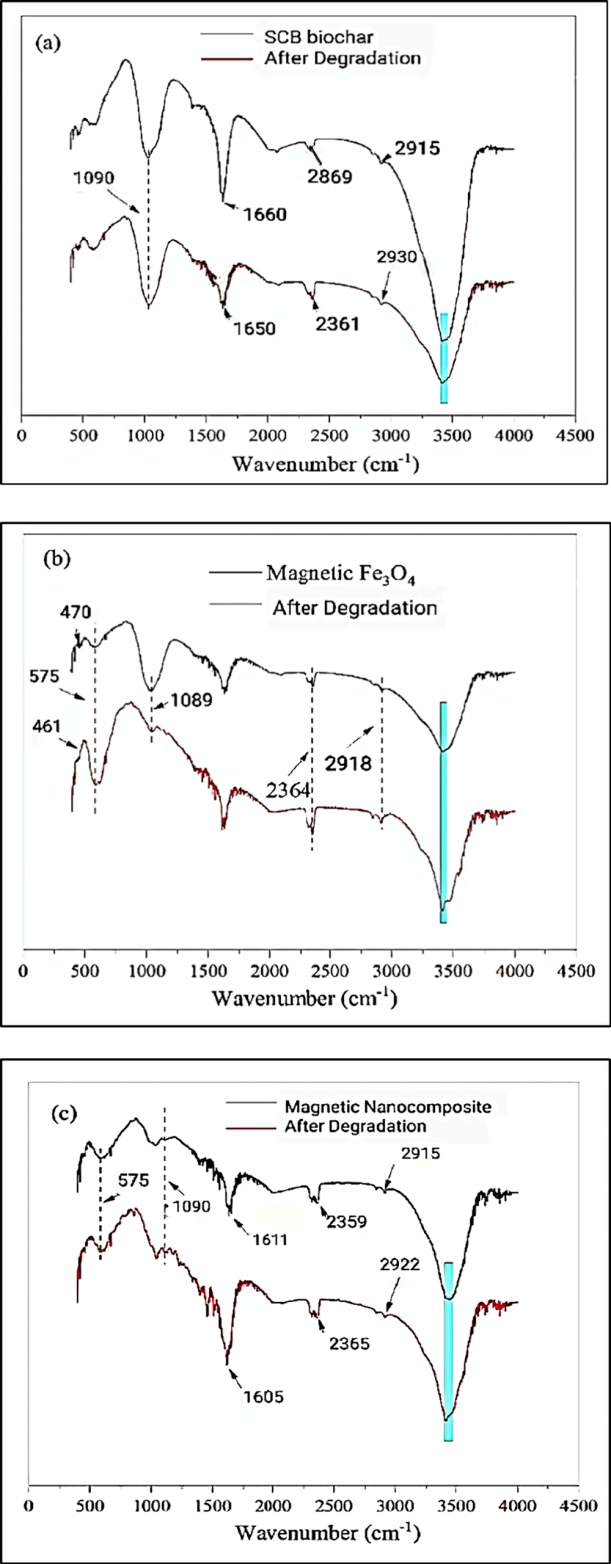
FT-IR spectra of biochar, magnetic NPs, and magnetic nanocomposite before and after the process of degradation. (a) Biochar, (b) Fe_3_O_4_ NPs, and (c) magnetic nanocomposite.

The FT-IR spectra displayed in Figure 8 illustrate the distinctive characteristics of biochar, magnetic NPs, and the magnetite/SCB biochar nanocomposite, complementing the structural information obtained from SEM, TEM, and EDX analyses. In SEM and TEM (Figure 5), the cluster-like morphology observed aligns with the FT-IR findings, indicating the successful integration of Fe_3_O_4_ NPs onto the biochar substrate. The emergence of an Fe–O vibration peak at 575 cm^–1^ in the FT-IR spectra corresponds to the iron (Fe) peaks identified in the EDX analysis. In particular, the stretching vibrations associated with C–O and C=O functionalities manifest as prominent peaks in the biochar spectrum at 1090 and 1660 cm^–1^, respectively. These observations indicated the presence of numerous oxygen-containing functional groups and aromatic rings within the biochar structure. Upon degradation, a noteworthy shift in the C=O stretching vibration peak is observed, transitioning from 1660 to 1650 cm^–1^. This shift signifies the active involvement of the C=O bonds in the degradation reaction. Additionally, the hydroxyl group (OH^−^) stretching vibration, which plays a vital role in the degradation of antibiotics, exhibits a remarkable reduction, manifesting as a degradation-induced peak at 3414 cm^–1^.^66^

This observation underscores the direct involvement of the hydroxyl groups in the degradation process. Furthermore, the aromatic rings within the biochar structure act as electron donors, establishing electron donor–acceptor interactions with the antibiotics. This interaction mechanism highlights the intricate nature of the degradation process, where the biochar’s aromatic rings play a key role in facilitating the degradation of antibiotics through electron transfer phenomena.^59,67^ The merging of magnetic Fe_3_O_4_ NPs with magnetic biochar leads to a discernible transformation in the FT-IR spectrum. Specifically, an intriguing peak emerges at 575 cm^–1^, credited to a distinctive Fe–O vibration. This spectral feature provides compelling evidence that magnetic NPs Fe_3_O_4_ has been successfully integrated onto the biochar substrate, signifying a fundamental step in the composite formation.

Moreover, this spectral alteration also hints at the interaction between the antibiotics and the ferrite group. This interaction appears to entail a complex interplay of metal ions, possibly representing a bridging mechanism. Remarkably, the peak intensities associated with both the magnetic Fe_3_O_4_ NPs and the Fe–O vibrations within the magnetic biochar undergo varying degrees of modification during the degradation process. These nuanced changes in spectral characteristics shed light on the dynamic nature of the interaction between antibiotics and the composite, suggesting a multifaceted interplay that merits further investigation.

In the realm of magnetic Fe_3_O_4_, the degradation process is orchestrated by distinctive vibrational phenomena. Notably, the stretching vibrations of C–O at 1090 cm^–1^ and C–H at 2915 cm^–1^ underpin the degradation peak. Meanwhile, the telltale sign of the OH^–^ stretching vibration is encapsulated within the 3400 cm^–1^ peak. This ensemble of functional groups appears to owe its presence to scrupulous surface cleaning of the magnetic Fe_3_O_4_ using ethanol and purified water during its production, inadvertently leaving traces of ethanol ensconced within the material’s internal pores. The magnetic biochar, under the influence of degradation, exhibits intriguing transformations in its vibrational spectra. First and foremost, the C–O stretching vibration peak at 1090 cm^–1^ registered an increase following degradation, signifying its active involvement in the degradation mechanism. Furthermore, the stretching vibrations of C=C and C=O experience discernible shifts from 1610 to 1606 cm^–1^, while the −C–H– stretching vibration shifted from 2915 to 2922 cm^–1^. These transitions unequivocally point to the meaningful participation of these functional groups in the degradation process. Remarkably, a broad absorption peak emerges at a wavelength of 3400 cm^–1^, intensifying as the degradation process advances. This spectral feature predominantly summarized the stretching vibration peak of OH^–^, emphasizing the essential role played by hydroxyl groups as prime sites for antibiotic degradation. In sum, the intricate interplay of these vibrational phenomena unveils the multifaceted nature of the degradation process, underscoring the significance of these functional groups in the transformation and ultimate removal of antibiotics.^2,68^

### 3.2 Optimization of Process Parameters

#### 3.2.1 Effect of pH on Photocatalytic Degradation of CIP and AMX

The pH of a solution is a significant factor for the degradation of antibiotics as it affects the solubility of the drug in the solution. The study used photocatalysts such as magnetic NPs, SCB biochar, and magnetic/SCB biochar nanocomposite to study the degradation of CIP and AMX. The results showed that the percentage of degradation decreased as the pH of the solution increased. The highest level of degradation efficiency for CIP was attained at pH 6, with percentages of 48.59% for NPs, 62.87% for biochar, and 73.03% for the nanocomposite. The photocatalysts showed better performance when tested under lower pH conditions, as evidenced by the magnetic nanocomposites achieving degradation rates of 73 and 57% at pH 6 and 10, respectively. Biochar exhibited substantial degradation capabilities, with degradation levels of 75 and 67% observed for CIP at pH 2 and 12, respectively. NPs follow the trend, i.e., 48% degradation at pH 6 and 31% degradation at pH 10.

**Figure 9:**
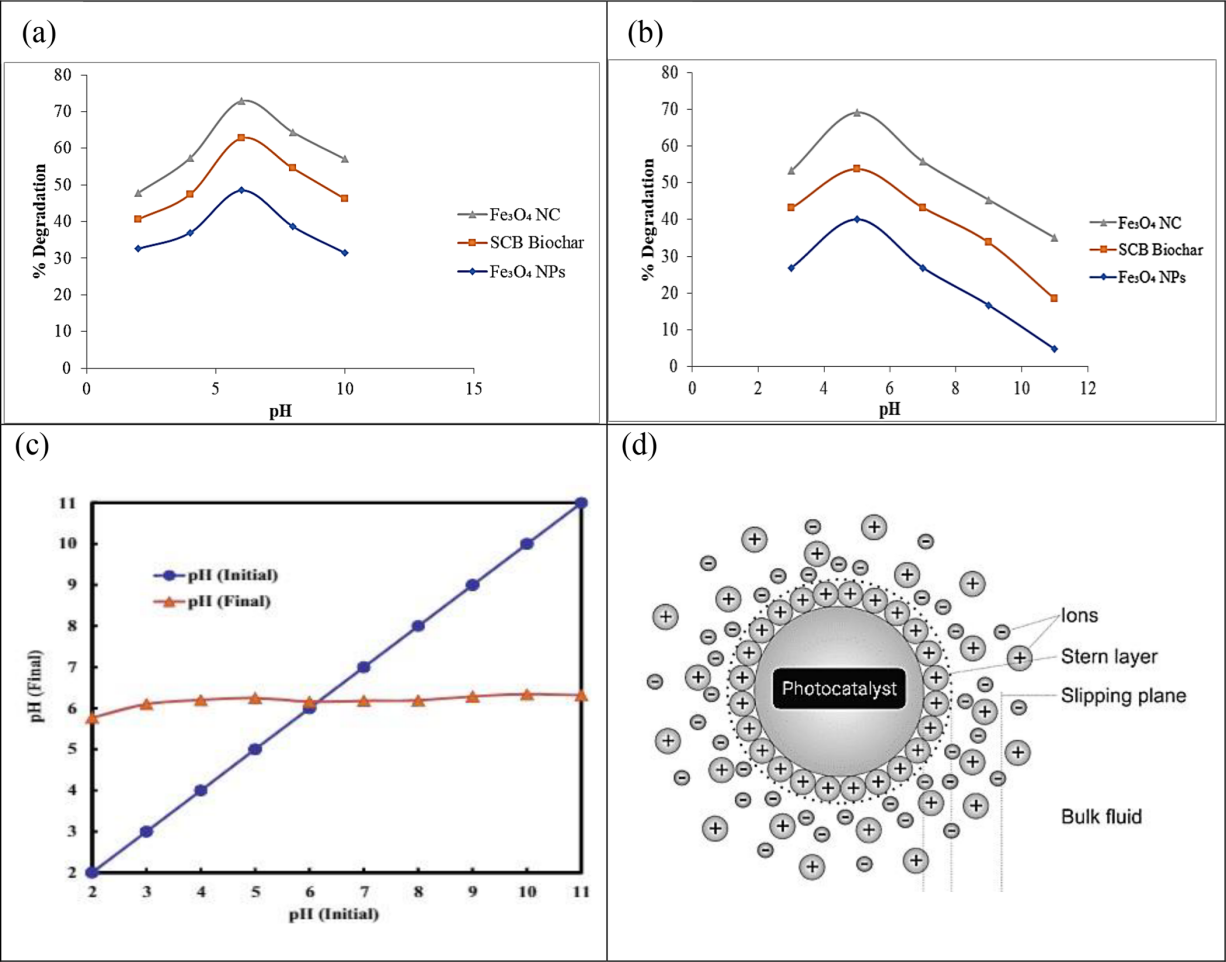
Effect of pH on photocatalytic degradation of (a) ciprofloxacin and (b) amoxicillin and pH point of zero charge (c, d).

The degradation efficiency of AMX was also determined using the pH as a factor. The highest degradation efficiency of AMX was attained at pH 5, with values of 39.99, 53.80, and 69.23% observed for three distinct photocatalysts, magnetic NPs, SCB biochar, and magnetic/SCB biochar nanocomposite, respectively. The degradation efficiency increased by increasing pH from 3 to 5, and the percentage degradation decreased with the increase in pH from 5 to 11. The maximum efficiency was obtained at pH 5. At a lower pH, the concentration of protons is high, so the drug’s surface becomes negatively charged, leading to higher degradation. At higher pH concentrations of hydroxyl radicals, repulsion occurs between the negatively charged drug surface and hydroxyl radicals, resulting in a decreased level of drug degradation. In simpler terms, the drugs can break down more easily when it is in an acidic (low pH) environment because it becomes negatively charged. However, when it is in a more basic (high pH) environment with lots of hydroxyl radicals, these troublemaking particles repel the medicine’s negative charge, making it harder for the medicine to break down. Figure 9 depicts the effect of pH on the degradation of CIP and AMX antibiotics.

The photocatalyst was found to have a pH point of zero charge (pHpzc) of 6, which elucidated its dynamic surface behavior. Below this pH, the positively charged surface facilitated stronger electrostatic interactions with ciprofloxacin and amoxicillin, reaching peak degradation efficiencies at pH 6 and 5, respectively. This insight into pH-dependent performance underscores the significance of pH control in optimizing the photocatalyst’s positive surface charge for enhanced adsorption and subsequent degradation of antibiotics in wastewater treatment applications. The effect of pH on photocatalytic degradation of (a) ciprofloxacin and (b) amoxicillin and pH point of zero charge (c, d) is presented in Figure 9.

#### 3.2.2 Effect of the Initial Concentration

The ensuing research study investigated the influence of the initial concentration on the degradation of CIP in aqueous solutions at varying concentrations. The initial concentration parameter is linked to the catalytic nature and functional group availability. Magnetic NPs, SCB biochar, and the magnetite/SCB nanocomposite are enriched in various functional groups. After 240 min, the percentage degradation of CIP exhibited an increment, with magnetic NPs demonstrating a degradation rate of 37.40% at a concentration of 60 mg/L, while biochar displayed a degradation rate of 47.21% at the same concentration. Remarkably, the nanocomposite exhibited superior performance attributed to its enhanced surface area and magnetic properties, resulting in a degradation rate of 62.23% at 60 mg/L.

**Figure 10:**
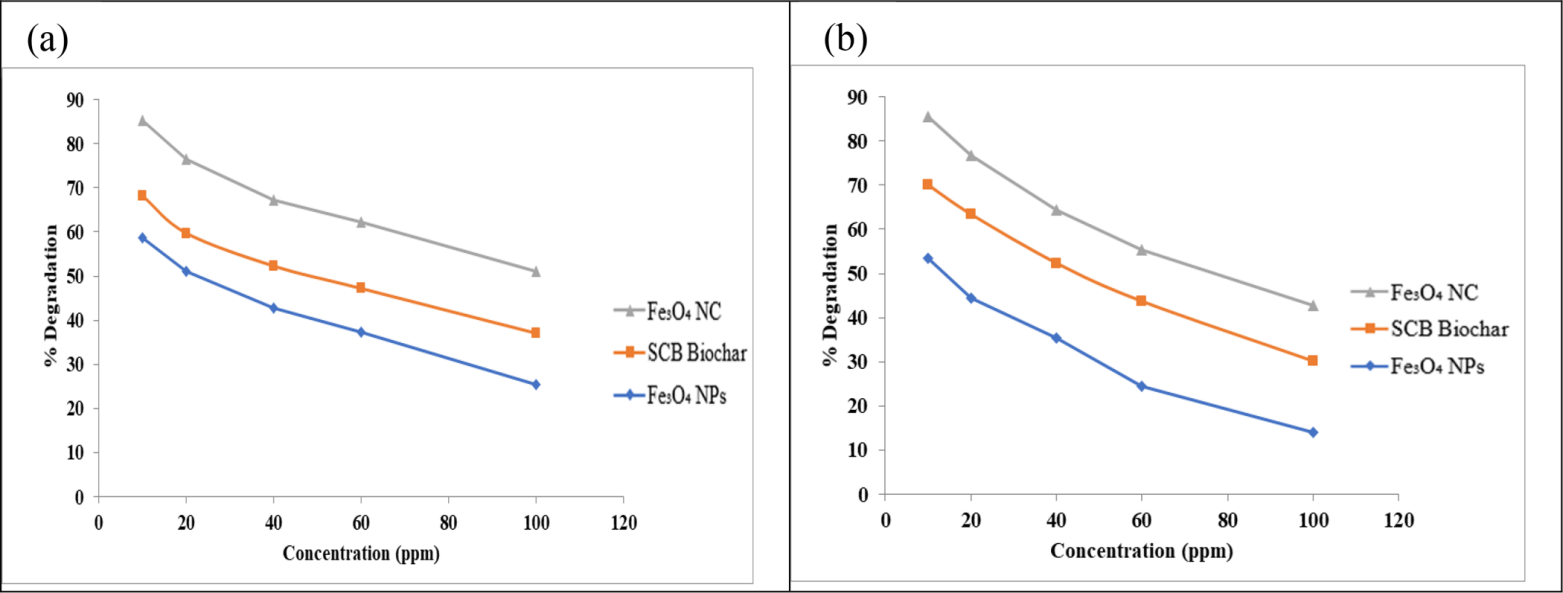
Effect of the initial concentration on the photocatalytic degradation of (a) ciprofloxacin and (b) amoxicillin.

The study also examined the influence of the initial concentration on AMX solutions under alkaline conditions (pH 11) by applying a photocatalyst dosage of 0.2 g. The magnetite nanocomposite proved to be a good catalyst for AMX. The maximum degradation of AMX obtained at a 10 mg/L concentration was in the order of the magnetic nanocomposite (85.55) > biochar (63.45) > NPs (53.34). The degradation of both drugs is concentration-dependent, and the degradation decreases with the initial concentration of the photocatalyst. At higher concentrations, there is competition for engaging the active sites, resulting in fewer photons reaching the photocatalyst surface and reducing the generation of electron–hole pairs and hydroxyl radicals. If the initial concentration is low, then it makes it easy to obtain a balance. So, it was observed that the degradation of drugs decreased with the increment of the catalyst concentration. Figure 10 illustrates the effect of the initial concentration on the degradation of CIP and AMX.

#### 3.2.3 Effect of Irradiation Time

**Figure 11:**
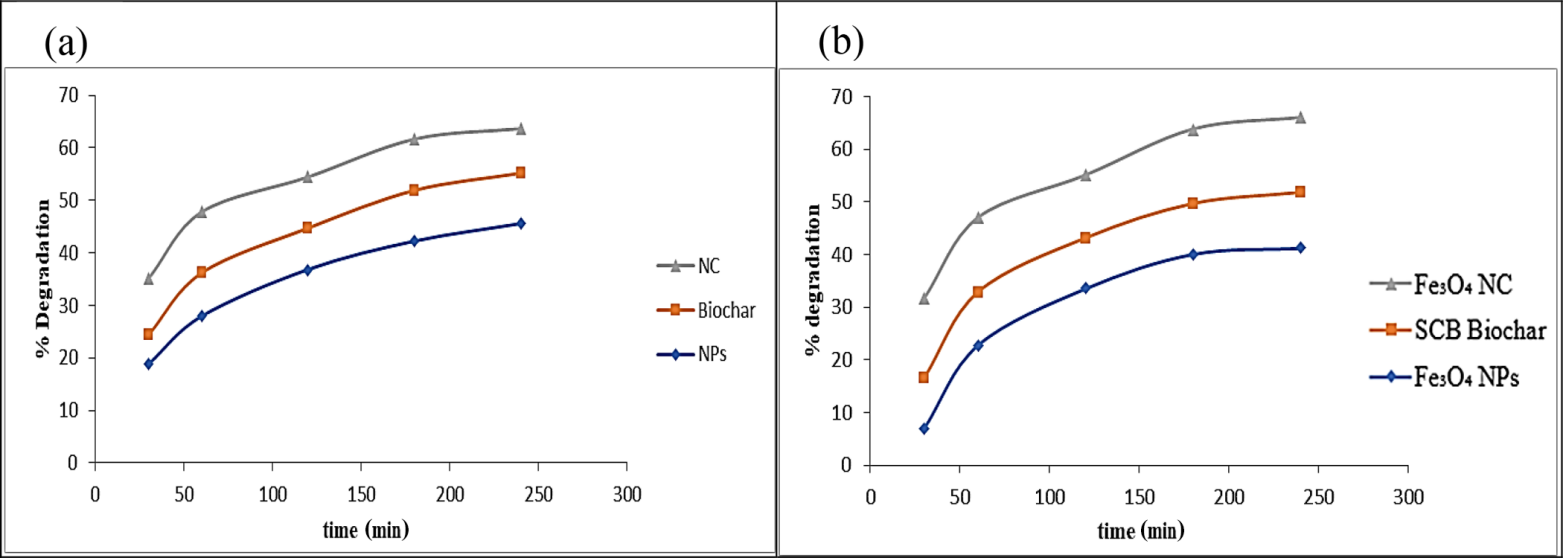
Effect of irradiation time on the photocatalytic degradation of (a) ciprofloxacin and (b) amoxicillin.

The irradiation time between the photocatalyst and contaminant is another important parameter that affects the performance of the photocatalytic degradation process. Through magnetic NPs, SCB biochar, and magnetite/SCB nanocomposite, the degradations of CIP at 30, 60, 120, 180, and 240 min were studied. The maximum degradation percentages were achieved at 240 min by applying three different catalysts; magnetic NPs, biochar, and magnetic nanocomposite were 45.49, 55.09, and 63.74% for CIP and 41.24, 51.78, and 65.97% for AMX, respectively. The findings indicated that an increase in irradiation time correlates with a proportional increase in the degradation percentage of drugs. The longer the drug must be in irradiation with the photocatalyst, the more likely it is to be degraded by the photocatalyst. Drug degradation increases with an exposure time of up to 240 min due to the increase in the free radicals (·OH) and degradation efficiency. At a 240 min irradiation time, the highest degradation of CIP was achieved. Drug degradation initially increases due to the higher driving force facilitating the transfer of the molecules of the drug to catalyst surfaces and active sites. After this period, the degradation rate decreases due to the reduction of the remaining active catalyst sites and the long-range diffusion effect of the molecules of the drug.^69^Figure 11 quantitatively shows the percentage degradation of CIP and AMX upon exposure.

#### 3.2.4 Effect of Photocatalyst Dosage

**Figure 12:**
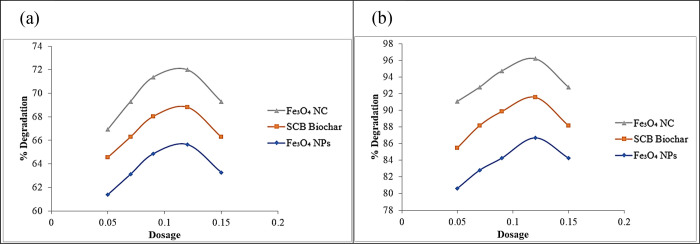
Effect of photocatalyst dosage on the photocatalytic degradation of (a) ciprofloxacin and (b) amoxicillin.

The percentage degradation of antibiotics increased as the catalyst dosage increased. The maximum degradation percentages were achieved by applying three different catalysts; magnetic NPs, SCB biochar, and magnetic/SCB biochar nanocomposite were 65.65% > 68.82% > 72.00% for CIP and 68.08% > 71.92% > 75.56% for AMX, respectively (Figure 11). Graphical results depicted that the nanocomposite showed better percentage degradation, i.e., 71–75% from a range of 0.05–0.15 g. Similarly, magnetic NPs and SCB biochar followed the same trend. The degradation rate of antibiotics depends on the number of active catalytic sites and the dosage of photocatalysts. By increasing the photocatalyst dosage, the active sites on the catalyst surface also increased, which in turn increased the amount of hydroxyl (·OH) and superoxide (O_2_^.–^) radicals and degraded drug. Addition of a photocatalyst beyond optimal amounts may cause turbidity and decreased photon absorption, affecting degradation efficiency. Insufficient drug molecules may cause increased degradation, but the active site remains saturated after reaching the equilibrium point. Consequently, when the amount of photocatalysts increased, the degradation of the drug also increased. Nevertheless, it is essential to recognize that if the catalyst concentration surpasses its optimal level, then the degradation effect tends to diminish or exhibit inconsistent outcomes due to the overlapping and overcrowding of catalyst molecules.^70^ The graphical representation is given in Figure 12.

#### 3.2.5 Comparison among Different Photocatalysts

**Figure 13:**
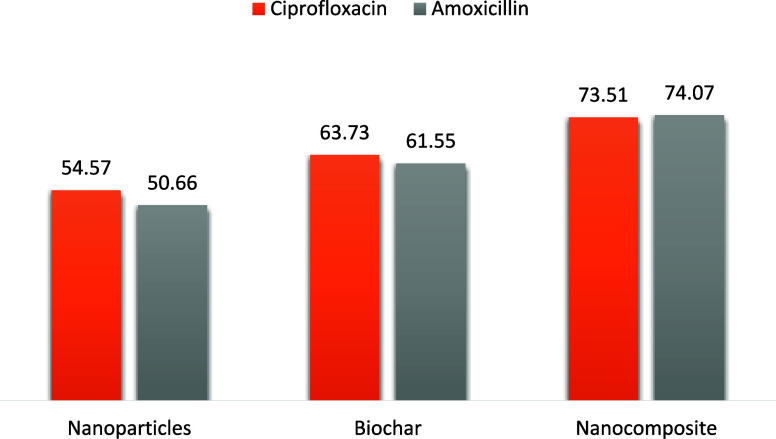
Comparison between different catalysts.

A comparative analysis was conducted under optimized conditions to assess the performance of the various catalysts. The study involved exposing 100 mg/L solutions of AMX and CIP to sunlight radiation for a duration of 2 h, with the addition of 0.2 g of NPs, biochar, and magnetic nanocomposite. It was observed that the optimal pH values for the degradation of AMX and CIP were 11 and 6, respectively. The concentration of the pharmaceuticals before and after degradation was determined by using a UV–vis spectrophotometer. The highest percentage of degradation for CIP was achieved in the following order: magnetic nanocomposite (73.51%) > biochar (63.73%) > NPs (54.57%). Similarly, AMX exhibited a similar trend in percentage degradation: nanocomposite (74.07%) > biochar (61.55%) > NPs (50.66%). The enhanced percentage degradation efficiency observed in the nanocomposites compared to NPs was due to the increased binding sites on the surface of biochar resulting from the incorporation of magnetic NPs.^1^ The degradation ability of NPs, biochar, and magnetic nanocomposites is depicted in Figure 13.

### 3.3 Kinetic Studies for the Degradation of Antibiotics

Through simple linear regression analysis, under different experimental conditions, the parameters of the kinetic model of degradation of CIP and AMX through the magnetic NPs, SCB biochar, and the magnetic/SCB biochar nanocomposite were calculated. The data of the first-order kinetic model is ln(*C*_e_/*C*_i_) vs *t*, for the second-order kinetic model, the graph is drawn between *t* and [(1/*C*_e_) – (1/*C*_i_)], and in the Behnajady–Modirashahla–Ghanbery dynamic model, the graph is drawn between *t* and *t*/1 – (*C*_e_/*C*_i_). In a remarkable pursuit of understanding the intricacies underlying the photocatalytic degradation facilitated by magnetite NPs, Malakootian et al. and Balarak et al. get on a scientific endeavor that pushes the boundaries of knowledge. Their approach involved the application of kinetic models to precisely analyze experimental data acquired at varying temperatures. The crux of their investigation revolved around dissecting the kinetics governing the degradation of AMX employing magnetite NPs. This work was executed through a scrupulous fitting of experimental data to a kinetic model established in previous studies, which aptly describes the process as a pseudo-first-order reaction. Key to this analysis was the determination of the observed rate constant, denoted as “*k*_obs_” and measured in units of (min^–1^). This crucial parameter was derived by plotting the natural logarithm of the ratio of the equilibrium concentration (*C*_e_) to the initial concentration (*C*_i_) against time (*t*) in min. The slope of this plot provided the elusive *k*_obs_, shedding light on the kinetics leading to the mesmerizing process of AMX degradation mediated by magnetite NPs.^30,35^

**Figure 14:**
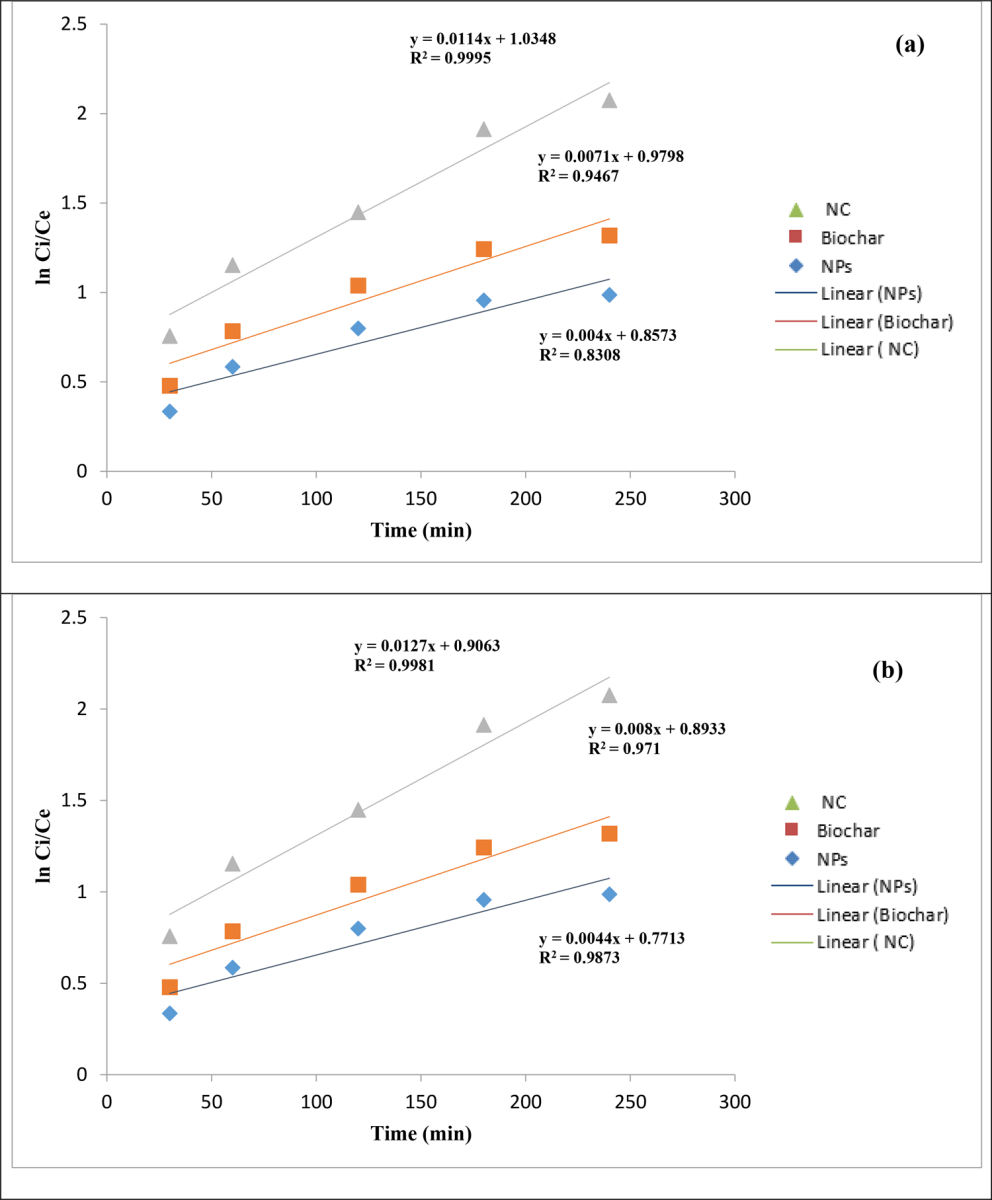
First-order plot of (a) ciprofloxacin and (b) amoxicillin.

**Figure 15:**
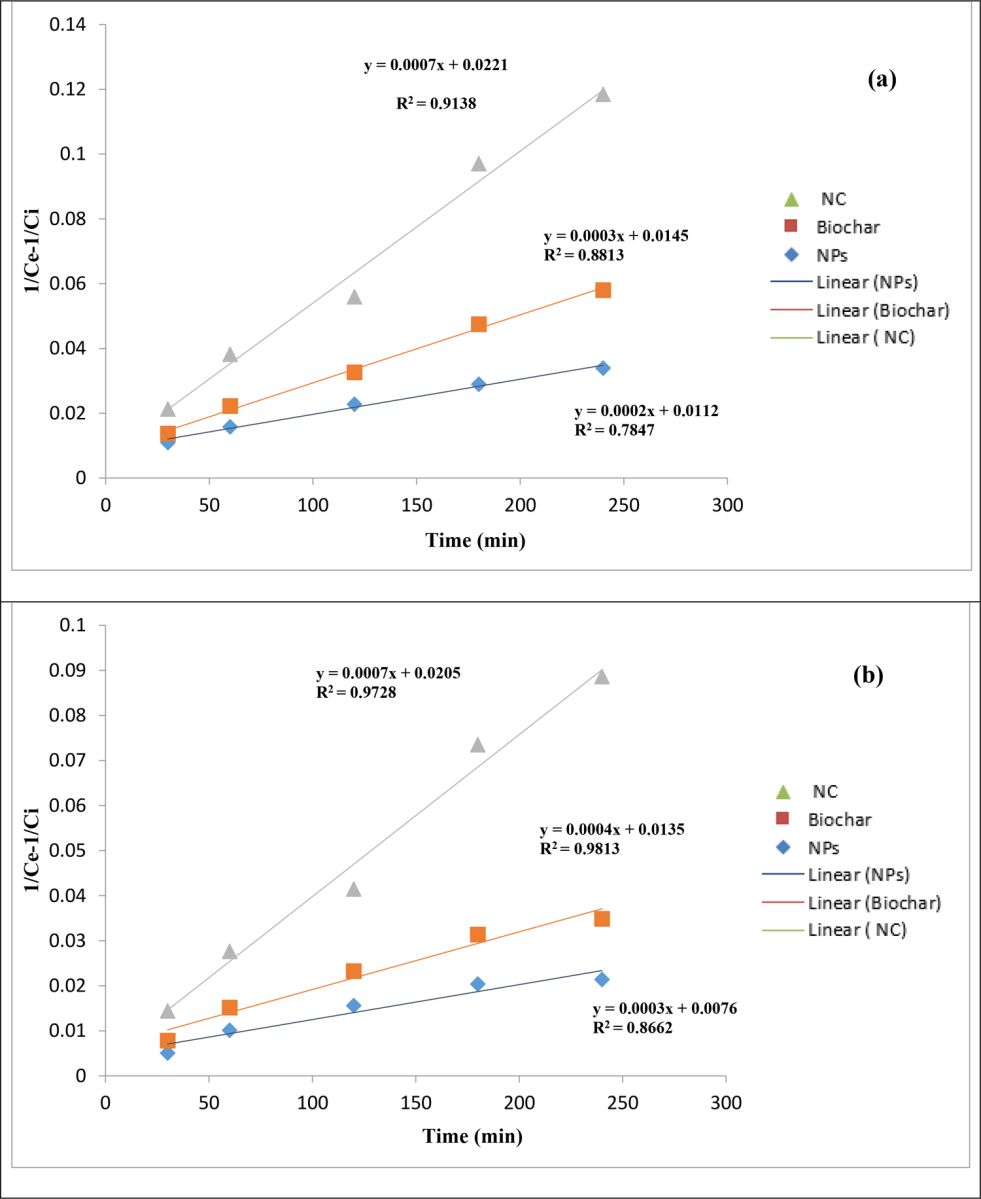
Second-order plots for (a) ciprofloxacin and (b) amoxicillin.

**Figure 16:**
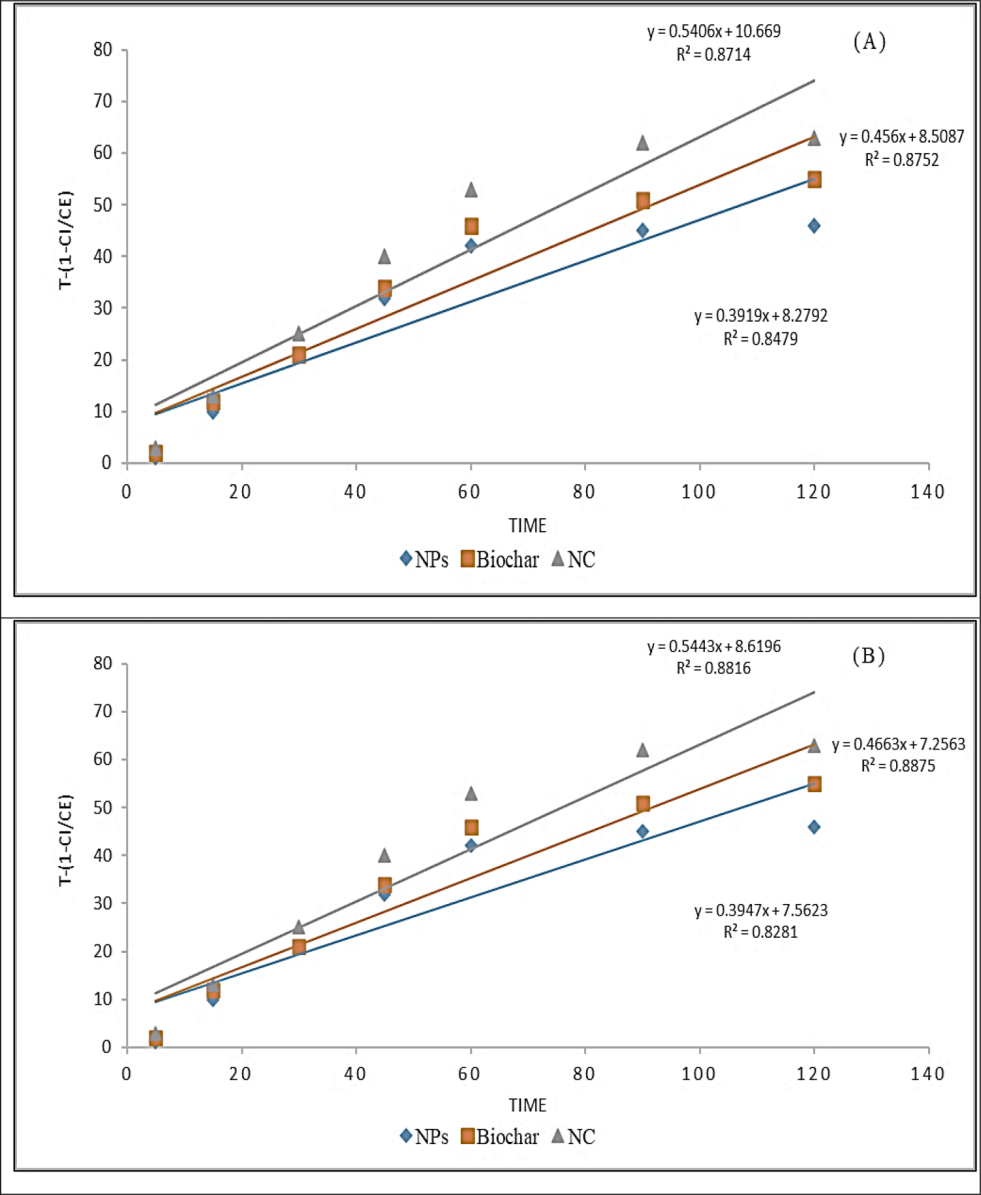
BMG for (A) ciprofloxacin and (B) amoxicillin.

The kinetics of CIP degradation were fastidiously assessed employing the pseudo-first-order model. The obtained correlation coefficients (*R*^2^) for concentrations of 3, 5, 7, and 9 mg/L were exceptionally high, measuring 0.905, 0.919, 0.921, and 0.930, respectively. These values reflect a robust alignment of the experimental data with the pseudo-first-order kinetics, underscoring the reliability of this model for describing the degradation process. To further illuminate the temperature-dependent nature of CIP degradation, regression analysis was conducted by plotting the observed rate constants (*k*_obs_) against temperature. The striking result was a coefficient of determination (*R*^2^) exceeding 0.957, an indication of the exceptional agreement between the degradation rates of CIP and temperature with a strong adherence to the pseudo-first-order kinetics model. These findings elucidated the key role of temperature in controlling the degradation kinetics of CIP. As temperature increases, the rate of degradation for CIP molecules intensifies, leading to the remarkable outcomes observed in our study. This temperature-dependent behavior holds significant implications for understanding and optimizing the degradation process of CIP in practical applications. First- and second-order plots for both antibiotics, CIP and AMX, are shown in Figures 14 and 15, respectively, and the BMG dynamic model graph for CIP and AMX is depicted in Figure 16.

The value of *R*^2^ for AMX and CIP, pseudo-first-order reaction, was in the order of 0.830 < 0.946 < 0.999 and 0.987 < 0.971 < 0.998 for magnetic NPs, biochar, and nanocomposites, respectively. It can be seen that the BMG and second-order kinetic models do not fit in the experimental data well, as indicated by the low values of the coefficients of determination. However, the value of the first-order determination coefficient is greater than the values of the BMG determination coefficient and the second-order kinetic model. Therefore, first-order kinetics is the best method to describe the degradation of AMX and CIP during the degradation process. Similar plots for kinetic studies are reported in literature.^19,20,46,49^ The obtained values of the rate constant and *R*^2^ are given in Table 1.

**Table 1:** Comparison of the First, Second, and BMG Models for AMX and CIP

		pseudo first order		pseudo second order				
		*K*_1ad_		*K*_2ad_		BMG		
drugs	photocatalyst	(min–1)	*R*^2^	(mg/min)	*R*^2^	1/m	1/b	*R*^2^
amoxicillin	Fe_3_O_4_NPs	0.0299174	0.830	0.0014	0.784	0.120	2.55	0.847
	biochar	0.0004342	0.946	0.0028	0.881	0.117	2.19	0.875
	Fe_3_O_4_NC	0.0004342	0.999	0.0031	0.913	0.093	1.85	0.871
ciprofloxacin	Fe_3_O_4_NPs	0.0037343	0.987	0.0028	0.866	0.132	2.53	0.828
	biochar	0.0048632	0.971	0.0021	0.972	0.137	2.14	0.887
	Fe_3_O_4_NC	0.0048798	0.998	0.0030	0.981	0.116	1.83	0.881

### 3.4 Recycling Test

**Figure 17:**
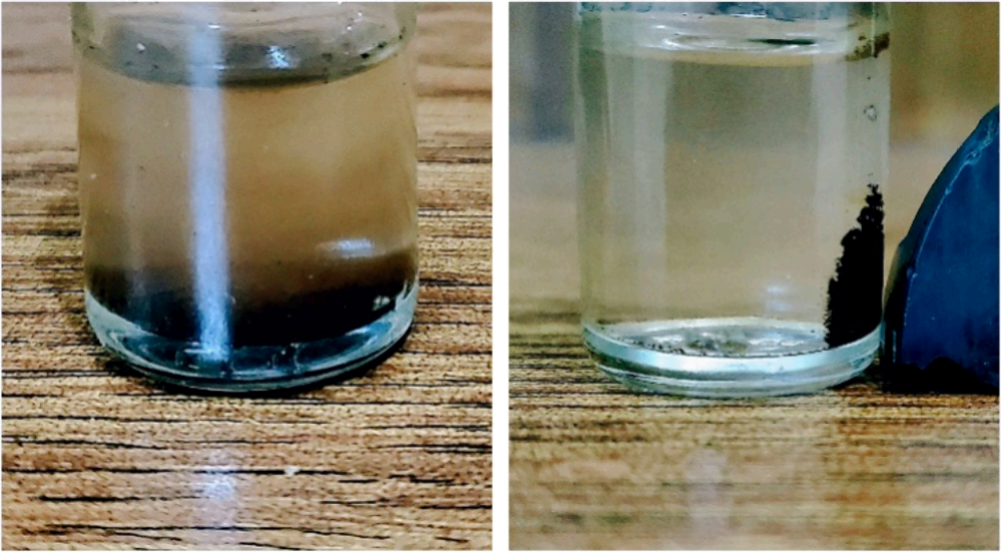
Magnetic separation of the photocatalyst.

After a 30 min light-driven reaction, the catalyst was spun and dried and then subsequently reintroduced into a 100 mL solution of antibiotics (10 mg L^–1^) for another round of testing to see if it could be reused. This worked well because the magnetite nanocomposite responds effectively to visible light, breaking down pollutants efficiently (Figure 17). It was found that it can be used again by exposing it to light after each round, and 30 min of light exposure proved optimal for removing stuck antibiotics. After magnetically separating it from the solution, drying it, and repeating this process three times, we noticed no significant loss of effectiveness, indicating that the magnetic nanocomposites remain stable and reliable for multiple uses in cleaning up pollutants.^33,71^ Conclusively, the photocatalyst was successfully recycled by a magnetic separation technique (external magnet) with only a slight decrease (7.4%) in catalytic activity. Green synthesis improved the degradation efficiency, but it did not have any impact on the reusability of the photocatalyst.^72^

## 4 Conclusions

In conclusion, the magnetic nanocomposite, composed of pyrolyzed biochar of sugar cane bagasse (SCB) and magnetite nanoparticles in a 5:1 ratio, exhibited exceptional photocatalytic degradation with degradation percentages surpassing benchmarks from a previous study.^87^ The achieved degradation percentages of 73.51% for AMX and 63.73% for CIP surpassed established benchmarks, underscoring the nanocomposite’s superiority (Table 2). By meticulously investigating parameters such as solution pH, dose rate, irradiation time, and initial catalyst concentration, our findings provide valuable insights for optimizing future applications. The calculated *R*^2^ values of 0.999 for AMX and 0.998 for CIP reflect the nanocomposite’s perfect fit to the pseudo-first-order reaction model, emphasizing its reliability across diverse experimental conditions. Significantly, the magnetite/SCB biochar nanocomposite emerged as the most effective catalyst, outperforming others in the photocatalytic degradation of antibiotics, as the addition of biochar prevented the agglomeration of photocatalytic nanoparticles and promoted the mobility of charge carrier. This outcome positions the nanocomposite as a benchmark-setting catalyst with quantitatively superior performance, advancing the field of photocatalysis for water treatment.

**Table 2:** Comparison of Activity Performance in This Work with Findings in the Existing Literature

parameter/aspect	experimental findings	literature findings
catalyst composition	magnetite/SCB nanocomposite, biochar, and magnetite NPs	iron oxide nanoparticles^73^
synthesis method	ecofriendly and sustainable	green synthesis^74^
		coprecipitation^75^
reducing agent	ferric chloride hexahydrate and *E*. *globulus* leaf extract	*Murraya koenigii* leaf extract (76)
photocatalyst application	photocatalytic degradation of ciprofloxacin and amoxicillin	photocatalytic degradation of tetracycline^73^
		ciprofloxacin^77^
		amoxicillin^41^
optimum conditions	pH (ciprofloxacin: 6, amoxicillin: 5), dosage (0.12 g), concentration (100 mg/L), and irradiation time (240 min)	for ciprofloxacin pH (3–11), reaction time (30–180 min), and catalyst loading (0.03–0.12 g).^78^
		for AMX nanoparticle dose (0.25–2 g/L), reaction time (10–120 min), and initial concentration (25–200 mg/L)^30^
comparative degradation efficiency of photocatalysts for CIP and AMX	magnetic nanocomposites (73.51 and 74.07%), biochar (63.73 and 61.55%), and magnetic NPs (54.57 and 50.66%)	80.74% degradation of CIP with CuFe_2_O_4_@methylcellulose (MC) nanophotocatalyst^79^
		γ-Fe_2_O_3_/Bi_2_WO_6_ (65%) nanocomposite^80^
		(∼50%) of the CIP was observed at pH 10 with ZnO nanoparticles^42^
		(77.01%) degradation of CIP with biochar supported the bismuth ferrite nanocomposite^81^
kinetic studies models applied	pseudo first order	pseudo first order
	pseudo second order	pseudo second order
	Behnajady–Modirashahla–Ghanbery	Behnajady–Modirashahla–Ghanbery application^43,82^
best kinetic model results	pseudo first order (all catalysts)	pseudo first order fitted best for degradation of AMX and CIP^30,81^
efficiency of percentage degradation (best results)	magnetic nanocomposites (amoxicillin: 90.88%, ciprofloxacin: 93.48%), biochar (amoxicillin: 79.43%, ciprofloxacin: 84.38%), and magnetic NPs (amoxicillin: 71.09%, ciprofloxacin: 74.75%)	(87.1%) maximum percentage degradation of CIP with GO-Fe_3_O_4_^83^
		maximum degradation (80%) of AMX with 30 mg/L dosage of TiO_2_^84^
		max degradation of 91.2% of CIP by reed biochar supported TiO_2_^85^
*R*^2^ values for pseudo first order (amoxicillin)	magnetic NPs (0.830), biochar (0.946), and nanocomposites (0.999)	reported coefficient of regression (*R*^2^) was 0.87 with AMX^84^
*R*^2^ values for pseudo first order (ciprofloxacin)	magnetic NPs (0.987), biochar (0.971), and nanocomposites (0.998)	*R*^2^ predicted = 0.70 for CIP with the BiFeO_3_ nanocomposite^34^
conclusion	the magnetite/SCB biochar nanocomposite showed the best results	the nanocomposite showed the best results^86^

## Supporting Information Available

Figure S1. Calibration curve of ciprofloxacin; Figure S2. Calibration curve of amoxicillin; Table S1. Data for the calibration curve of ciprofloxacin at λ_max_ of 238 nm. Table S2. Data for the calibration curve of amoxicillin at λ_max_ of 341 nm (PDF)
