# Supplementary material for: Photocatalytic Degradation of Antibiotics via Exploitation of a Magnetic Nanocomposite: A Green Nanotechnology Approach toward Drug-Contaminated Wastewater Reclamation
Source: ACS Omega. 2024 Feb 6;9(7):7986–8004. doi: 10.1021/acsomega.3c08116 (PMC10882661; doi:10.1021/acsomega.3c08116)
Supplement: Supplementary file 1 — ao3c08116_si_001.pdf [file ao3c08116_si_001.pdf]

# Electronic Supplementary Information

# Photocatalytic degradation of antibiotics via exploitation of magnetic nanocomposite: A green nanotechnology approach towards drug-contaminated wastewater reclamation

Noor Zulfiqar[1*], Raziya Nadeem[2] Othman AI Musaimi[3&4]

[*1]Department of Chemistry, Faculty of Science, University of Agriculture, Faisalabad, Pakistan

[2]Department of Chemistry, Faculty of Science, University of Agriculture, Faisalabad, Pakistan

[3]School of Pharmacy, Faculty of Medical Sciences, Newcastle upon Tyne, UK NE1 7RU, UK

[4]Department of Chemical Engineering, Imperial College London, London SW7 2AZ, UK

*Correspondence: Email: 2018ag3898@uaf.edu.pk; Tel: +923178354635

## The Supplementary Material file includes

**Figure S1.** Calibration curve of Ciprofloxacin

**Figure S2.** Calibration curve of Amoxicillin

**Table S1.** Data for calibration curve of Ciprofloxacin at $\lambda_{max}$ of 238 nm

**Table S2.** Data for calibration curve of Amoxicillin at $\lambda_{max}$ of 341 nm

## Calibration curves of drugs

The calibration curve of Ciprofloxacin and Amoxicillin was plotted using different concentrations of Ciprofloxacin and Amoxicillin in water in the range of (10-100) mg/L. The photo degradation was measured at the maximum absorbance wavelength ($\lambda_{max}$) 277 nm and 341 nm for Ciprofloxacin and Amoxicillin respectively. The obtained value of $R^2$ is 0.9998 and 0.9989 for Ciprofloxacin and Amoxicillin respectively. The data and curve of Ciprofloxacin and Amoxicillin are shown in Table 1, 2 and Fig 1 and 2 respectively. A graph of straight line was obtained for the both antibiotics, Amoxicillin and Ciprofloxacin was plotted against concentration which shows that the absorbance was increased by increasing the concentration. Therefore, there is a direct relation between concentrations of analytes (Amoxicillin and Ciprofloxacin) and absorbance from Beer Lambert's law

$$A = €Cl$$

A = Absorbance

€ = extinction coefficient

C = Concentration

l = path length between sample holder and source

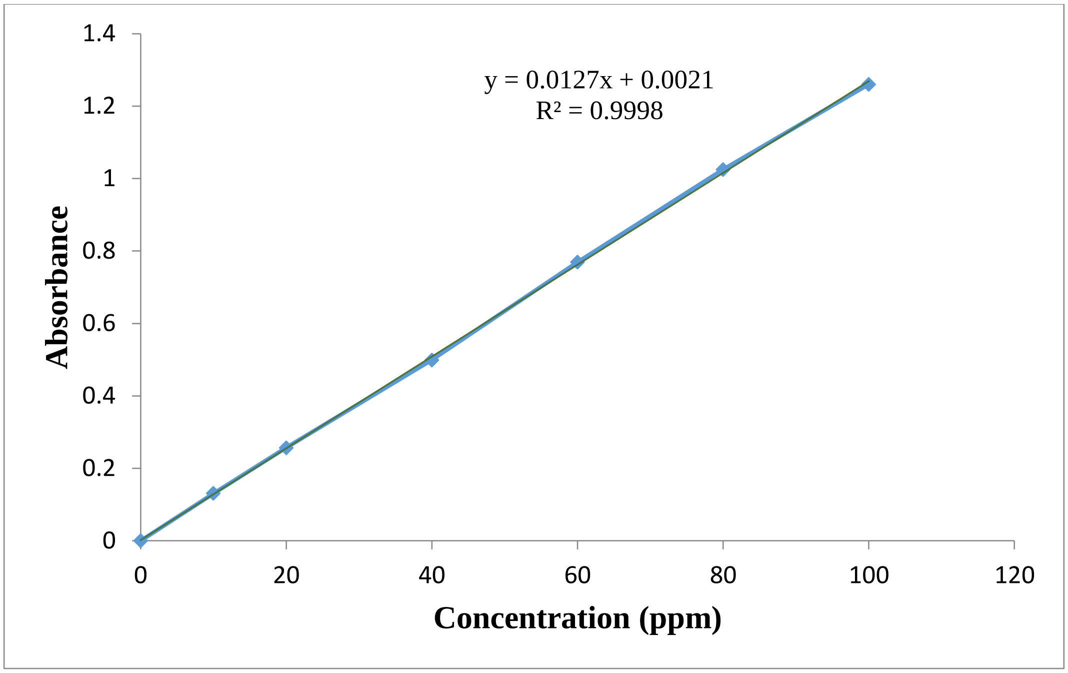


**Figure. S1. Calibration curve of Ciprofloxacin**

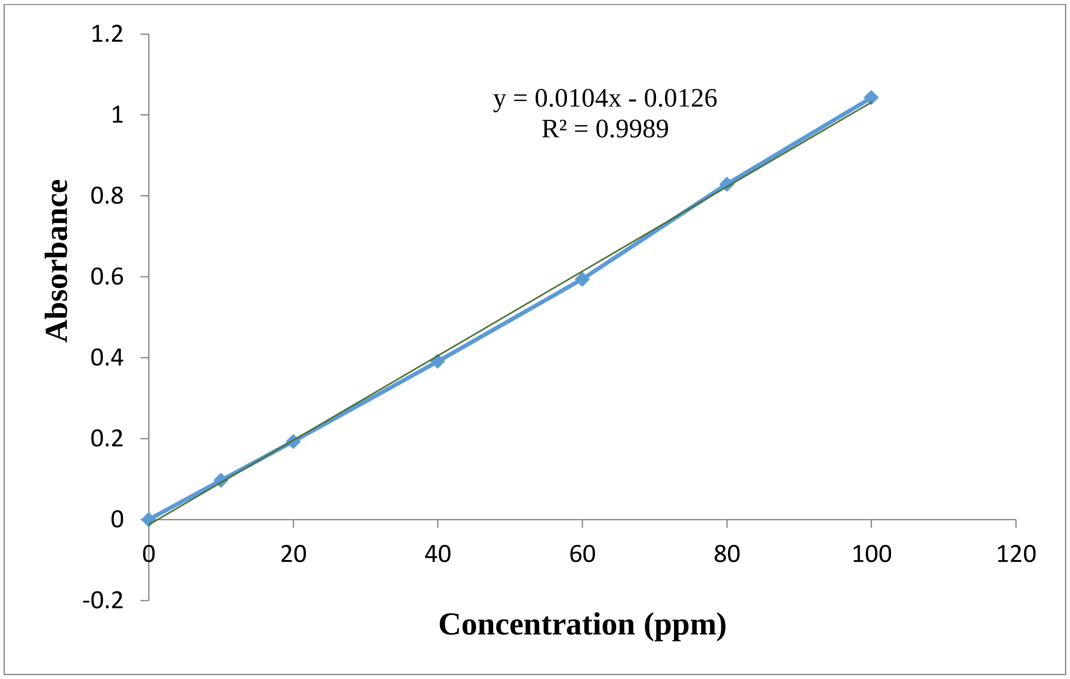


**Figure. S2. Calibration curve of Amoxicillin**

**Table S1. Data for calibration curve of Ciprofloxacin at $\lambda_{max}$ of 238 nm**

| Drug | Concentration | Absorbance |
|---|---|---|
| Ciprofloxacin | 0 | 0 |
| | 10 | 0.131 |
| | 20 | 0.257 |
| | 40 | 0.499 |
| | 60 | 0.769 |
| | 80 | 1.025 |
| | 100 | 1.26 |

**Table S2. Data for calibration curve of Amoxicillin at $\lambda_{max}$ of 341 nm**

| Drug | Concentration | Absorbance |
|---|---|---|
| Amoxicillin | 0 | 0 |
| | 10 | 0.097 |
| | 20 | 0.193 |
| | 40 | 0.391 |
| | 60 | 0.594 |
| | 80 | 0.829 |
| | 100 | 1.043 |
